# COVID-19 prevalence and mortality in longer-term care facilities

**DOI:** 10.1007/s10654-022-00861-w

**Published:** 2022-04-10

**Authors:** Andrew T. Levin, Juulia Jylhävä, Dorota Religa, Laura Shallcross

**Affiliations:** 1 Dartmouth College, Hanover, USA; 2 National Bureau for Economic Research, Cambridge, USA; 3 Center for Economic Policy Research, London, United Kingdom; 4 Department of Medical Epidemiology and Biostatistics, Karolinska Institutet, Stockholm, Sweden; 5 Faculty of Social Sciences, Unit of Health Sciences and Gerontology Research Center, University of Tampere, Tampere, Finland; 6 Department of Neurobiology, Care Sciences and Society, Karolinska Institutet, Stockholm, Sweden; 7 University College, London, United Kingdom

**Keywords:** SARS-CoV-2, seroprevalence, infection fatality rate, nursing homes, care facilities

## Abstract

This essay considers the factors that have contributed to very high COVID-19 mortality in longer-term care facilities (LTCFs). We compare the demographic characteristics of LTCF residents with those of community-dwelling older adults, and then we review the evidence regarding prevalence and infection fatality rates (IFRs), including links to frailty and some comorbidities. Finally, we discuss policy measures that could foster the physical and mental health and well-being of LTCF residents in the present context and in potential future pandemics.

*“Yet somehow our society must make it right and possible for old people not to fear the young or be deserted by them, for the test of a civilization is in the way that it cares for its helpless members.“ Pearl S. Buck, Nobel Laureate for Literature*[1].

As the COVID-19 pandemic has surpassed the two-year mark, its overall impact on mortality has been devastating, as shown in Table 1. In effect, the cumulative death toll in 2020-21 comprised about 1/400th of the entire population of Great Britain and of the USA and about 1/600th of the population of Sweden. Moreover, it has been clear that COVID-19 is dangerous for younger and middle-aged adults, not merely the elderly and infirm.[2–4] In the USA, for example, younger and middle-aged adults have accounted for nearly one-half of the total years of life lost (YLL) from COVID-19. In Great Britain and Sweden, community-dwelling older adults have accounted for a majority of the total YLL. Nonetheless, the impact of the pandemic has been most severe for long-term care facility (LTCF) residents, whose COVID-19 mortality rates have been an order of magnitude higher than those of community-dwelling older adults.

In this essay we consider the factors that contributed to such high COVID-19 mortality rates in LTCFs. We begin by comparing the demographic characteristics of LTCF residents with those of community-dwelling older adults. Next, we review the evidence regarding COVID-19 prevalence and infection fatality rates (IFRs) in LTCFs, including links to frailty and some comorbidities. Finally, we consider public policy measures that could foster the physical and mental health and well-being of LTCF residents and reduce the impact of infectious diseases in the present context and in potential future pandemics.

**Table 1:** Patterns of COVID-19 Fatalities in 2020-21

	Great Britain	Norway	Sweden	USA
***Mortality Rate (per 100 K person-years)***				
Entire Population	132	12	76	125
Younger & Middle-Aged Adults	26	3	14	49
Older Adults	616	80	445	559
*Community-Dwelling*	479	35	288	441
*LTCF Residents*	4652	1170	3432	5370
***Share of Total Years of Life Lost (%)***				
Younger & Middle-Aged Adults	40	36	26	47
Older Adults	60	63	74	52
*Community-Dwelling*	50	40	56	43
*LTCF Residents*	10	23	18	9

*Note: Younger & middle-aged adults = ages 15–64 for Great Britain & USA, 20–69 for Norway & Sweden. Older adults = ages 65 + for Great Britain & USA, ages 70 + for Norway & Sweden. Sources: Norway FHI*[5], *Sweden PHA*[6], *Scotland NRS*[7], *UK ONS*[8, 9], *US CDC*[10] *and CMS*[11]; *see also France INED*[12]. *YLL reflects age and sex but not comorbidities.*[3, 13, 14]

## Demographic Characteristics of LTCF Residents

In characterizing LTCF residents as distinct from other “community-dwelling” older adults, it must be recognized that these terms do not have precise and uniform scientific definitions but instead reflect substantial variations in cultural norms and socioeconomic status as well as governmental programs and regulations. Indeed, LTCFs are exceedingly rare in developing countries, where older adults generally live with their children or other relatives. For high-income countries, the OECD defines LTCFs as residential facilities that provide health services at the level of nursing care, as distinct from hospitals (which can provide much higher levels of medical care) or other residential arrangements (which may offer a mix of personal care and other services).[23] It should be noted that the OECD uses the term *“elderly”* in describing adults ages 65+, whereas we simply refer to this demographic group as *“older adults.”*[24, 25].

As shown in Table 2, only a small fraction of older adults reside in LTCFs, but the proportion in Sweden is notably higher than in the UK and nearly twice that of Spain and the USA. These outcomes reflect a number of distinct factors:

- In all four countries, life expectancy (as of age 65) extends into the mid-80s. However, healthy average life expectancy – defined as the absence of any major disease – is typically about 5 years shorter than that, with varying degrees of healthcare and personal assistance that may be needed over the intervening period.
- The proportion of older adults (ages 65 + years) who live together with their children varies markedly across countries, ranging from 1·5% in Sweden to more than 30% in Spain. Such residential arrangements are likely to be associated with differences in physical and social activity that may be consequential for health and cognition.[15, 26] For example, the reported incidence of falls per person-year (a key indicator of frailty) is nearly twice as high in Sweden compared to Spain.
- The proportion of older adults receiving longer-term care at home differs substantially across countries, ranging from 8% in the USA to 12% in Sweden and the UK. These outcomes reflect individual preferences as well as public policies aimed at encouraging residence at home rather than in LTCFs whenever possible.[26, 27].
- Older adults face an array of residential options depending on income and health status, including retirement communities and group homes for those who can live independently, assisted living for those with specific needs or disabilities, care homes for the frail and cognitively impaired, skilled nursing facilities for those with severe illness, and hospices to provide end-of-life care.[21, 28].
- The prevalence and degree of frailty is strongly linked to age, with an incidence of severe frailty among community-dwelling adults of about 4% at ages 65–69, 10% at ages 75–79, and 27% at ages 85+.[29] A recent meta-analysis concluded that the overall incidence of frailty was about 50% for ambulatory individuals (mean age 81) and about 70% for LTCF residents (mean age 86), but severe frailty was much more frequent among the latter group.[30].

Generally speaking, health and mortality data for LTCFs encompasses elder care homes and skilled nursing facilities, whereas data regarding other residential arrangements has a lower degree of consistency and comparability. For example, the U.S. Center for Medicare and Medicaid Services oversees all U.S. nursing homes, while state agencies may regulate other types of facilities for older adults (such as assisted living and group homes). The U.S. National Center for Health Statistics also reports on long-term care provided by adult day service centers, home health agencies, and hospices.[31].

The duration of care in LTCFs varies widely depending on the individual’s health status and other factors. In the USA, for example, 14% of individuals in nursing homes are covered by Medicare, which reimburses up to 100 days of skilled nursing care following hospitalization.[31] More than one-third of U.S. nursing home residents have been diagnosed with heart disease, which can greatly foreshorten life expectancy. Nearly half of U.S. nursing home residents have been diagnosed with Alzheimer’s disease or other types of dementia, for which the post-diagnosis 5-year survival probability is about 50%.[32, 33] A study of U.K. care home residents found that the median length of stay was 1·6 years, with a 75th percentile of 3·6 years and a 90th percentile of 6·2 years.[34].

Importantly, as shown in Table 2, the median age of LTCF residents is markedly higher than that of the overall population of older adults: This age gap is about 9 years in the USA, 10 years in the UK, and 12 years in Spain and Sweden. Accounting for these age differences is crucial in comparing COVID-19 IFRs for LTCF residents vs. community-dwelling older adults.

**Table 2:** Demographic Patterns for Older Adults

Indicator	Spain	Sweden	UK	USA
Population Share of Older Adults, Ages 65+ *(%)*	20	20	19	16
Life Expectancy at Age 65	86	85	85	84
Healthy Life Expectancy at Age 65	81	80	80	79
Living with Children, Ages 65+ *(%)*	34	1.5	11	19
Rate of Falls at Ages 75–79 *(%)*	6	11	8	10
Long-Term Care at Home, Ages 65+ *(%)*	9	12	12	8
Long-Term Care Facility, Ages 65+ *(%)*	2·2	4·2	3·3	2·4
Median Age of All Adults Ages 65+	75	75	74	72
Median Age of LTCF Residents, Ages 65+	87	87	84	81

*Sources: World Health Organization, Global Burden of Disease, Sweden Institute, U.S. National Center for Health Statistics, Albertini et al.(2018), Ballin et al. (2021), Candel et al. (2021),Chudasama et al. (2021).*[15–22]

## COVID-19 Prevalence in LTCFs

Numerous epidemiological studies have found that the prevalence of COVID-19 in LTCFs was far higher than among community-dwelling older adults. For example, a serology study of northern Italy during spring 2020 identified anti-SARS-CoV-2 antibodies in about 50% of LTCF residents compared to about 11% of the general population.[35] The LTCF/community ratio of prevalence was even higher in other locations: 13x in a study of 179 UK care homes and about 30x in a study of LTCFs in Fulton County, USA.[36–38] These disparities reflect the extent to which community-dwelling older adults could effectively limit their potential exposure to the virus (getting home food deliveries, minimizing social contacts, etc.), whereas LTCF residents inevitably have frequent and direct physical contact with LTCF staff.[39] Moreover, about one-third of COVID-19 infections are asymptomatic, even in elderly adults.[40, 41].

Protecting LTCF residents was particularly difficult at the early stages of the pandemic when rapid tests and protective equipment were not readily available and methods of mitigating infection risk were not well-established. In such circumstances, a single LTCF staff member might inadvertently spread the virus to a large fraction of LTCF residents. Studies conducted in Scotland and in Ontario, Canada found that there were outbreaks in about 40% of LTCFs.[42, 43] A systematic review and meta-analysis found that about 45% of residents were infected in facilities experiencing an outbreak, and in the majority of such instances it could not be determined how the virus was introduced into the facility.[41].

## Assessing the Cause of Death

At an early stage of the pandemic, the WHO established guidelines for certifying COVID-19 as a confirmed cause of death (in cases with a positive test) or as a suspected cause of death (based solely on observed symptoms).[44] During 2020-21, the ratio of confirmed to total COVID-19 fatalities was 97% in England and 96% in Sweden.[6, 45, 46] Nonetheless, the precise application of the WHO guidelines has differed somewhat across countries, and hence caution is needed in making international comparisons. Moreover, implementing these guidelines is particularly complex in assessing fatalities of LTCF residents, who may have been extremely frail or had a severe preexisting illness.

Autopsies can be useful in determining causes of death but are quite unusual for elderly individuals. However, several peer-reviewed studies have reported on systematic autopsies of older adults to determine the frequency of cases in which COVID-19 was not a cause of death although the deceased had a confirmed positive test.[47–50] In these studies, COVID-19 was found to be a direct or secondary cause of death in the vast majority of PCR-positive individuals, regardless of whether the death occurred at home or at a hospital or LTCF. Diffuse alveolar damage was evident in nearly all instances in which COVID-19 was a causal factor of death, while thrombosis was observed in a large proportion of those cases.[47, 50] Autopsies have also documented some specific cases, such as carbon monoxide poisoning, where LTCF residents clearly died “with COVID” rather than “from COVID.”[51].

## Gauging Fatality Rates

The proportion of deaths among confirmed infections – commonly referred to as the *case fatality rate (CFR)* – can be used as an approximate gauge of the severity of COVID-19 in LTCFs.[41] However, numerous studies have documented the pitfalls of using CFRs, reflecting the incidence of asymptomatic COVID-19 infections that are never detected as well as constraints on the availability of rapid tests, especially during the early stages of the pandemic.[35, 38, 40, 52].

An alternative metric is the proportion of deaths among hospitalized COVID-19 patients, commonly referred to as the *hospitalization fatality rate (HFR)*. For the general population, most COVID-19 deaths have occurred in hospitals, and the HFR has generally been much higher than the CFR, reflecting the fact that hospital admissions involve individuals who are experiencing severe symptoms. By contrast, LTCF residents with COVID-19 have only rarely been transferred to a hospital. For example, a UK study found that only 4% of the COVID-19 fatalities of LTCF residents occurred in hospitals.[42].

The low frequency of hospitalization for LTCF patients reflects several distinct factors. First, LTCFs are able to provide significant medical treatment for COVID-19 (such as anticoagulants, antibiotics, and hydration), thereby avoiding a hospital transfer that could be quite traumatic for the patient. Second, some LTCF residents (or their legal guardian) may have signed a *“do not resuscitate”* order, signaling their preference for avoiding the use of invasive procedures (such as a mechanical ventilator) that would prolong the individual’s life. Finally, in some circumstances where hospital capacity is highly constrained, triage procedures may assign priority to younger and healthier patients while limiting the hospital admission of the aged and infirm.[53, 54] All of these factors create risk of bias that hampers the use of HFRs in drawing inferences regarding LTCF residents.

The gold standard in gauging severity is the number of deaths in proportion to the number of infected individuals, commonly referred to as the *infection fatality rate (IFR)*.[2] For example, a study in Georgia USA found an IFR of 18% for LTCFs that conducted periodic antigen tests of all residents and staff and an IFR of 15% for facilities where all residents were tested promptly after identification of an index case.[37] The Vivaldi serology study analyzed a large representative sample of care home residents in England and found seropositivity of 33% during the baseline period of summer and early fall 2020.[55] Thus, in the total population of about 425,000 care home residents, there were about 140,000 COVID-19 infections.[56] As of 30 October 2020, England reported 20,617 COVID-related fatalities of care home residents, of which more than 80% were in residents ages 80+.[8] In effect, the estimated IFR of LTCF residents in England was about 15%.

These estimates of IFR for LTCF residents are comparable in magnitude to the predictions of a prior metaregression analysis of age-specific IFRs published in this journal.[2] In particular, LTCF residents in Georgia USA have a median age of about 82 years, while LTCF residents in England have a median age of 86 years. The metaregression predicts an IFR of 15% (CI: 12–20%) for a comparable cohort with a median age of 85 years.

It should also be noted that the IFR for LTCF residents may have been extraordinarily high during the initial onset of the COVID-19 pandemic, due to incapacitation of facility staff and limited information about effective treatment methods as well as constraints on hospitalization and admission to intensive care units.[53, 54, 57] For example, at one small LTCF in southwestern France, an outbreak started on 14 March 2020 that infected half of the staff and two-thirds of the residents, whose case fatality rate was nearly 30%.[58] Similarly, an outbreak was identified on 17 March 2020 at a small LTCF in Ontario, Canada, infecting half of the staff and nearly all of the residents, whose case fatality rate was 45%.[59] In a study of Swedish LTCF residents (median age 87), the 30-day mortality during the first wave of the pandemic was about 40% for COVID-19 cases compared to about 6% for the control group.[16] In a comparable study of Ontario, Canada, the 30-day mortality for COVID-positive LTCF residents (median age 86) was 24% higher than that of the control group and moderately above the metaregression IFR prediction of 17% (CI: 14–22%) for that age cohort.[60, 61].

## Frailty and Comorbidities

Frailty has been linked to elevated COVID-19 mortality among LTCF residents, reflecting a combination of increased incidence of infection as well as more adverse consequences—the same combination that has been observed for other infectious diseases.[62, 63] Individuals with severe frailty or cognitive deficiencies require extensive assistance with daily routines and hence greater exposure if a staff person becomes infected. Moreover, frailty has a strong association with immune dysregulation that is likely to exacerbate vulnerability to COVID-19 as with other infectious diseases.[64, 65] However, a systematic review of the links between frailty and COVID-19 mortality found that nearly all studies have involved hospitalized LTCF patients; such findings are subject to elevated risk of bias for the reasons noted above.[66] That review identified two other studies involving samples of LTCF residents, but those findings were hampered by limited size and diagnostic methods.[67, 68] A large-scale study of UK Biobank participants found a strong association between frailty, hospitalization, and COVID-19 mortality in the overall sample but did not find a significant link between frailty and mortality within the subsample of hospitalized patients.[69].

COVID-19 mortality has also been independently associated with some specific comorbidities. Large-scale studies of LTCF residents with COVID-19 did not observe any significant association between 30-day mortality risk and certain chronic conditions (i.e., chronic obstructive pulmonary disease, hypertension, coronary artery disease, heart failure) but did identify moderately elevated odds ratios for diabetes (OR 1·2; CI: 1·1–1·4) and chronic kidney disease (OR 1·3; CI: 1·1–1·6).[16, 70] Mortality risk was linked more strongly to physical and cognitive impairments, although those links might at least partially reflect increased risk of exposure for the reasons noted above.[71].

## Comparison with Community-Dwelling Older Adults

Age differences are highly relevant in accounting for differences in estimated IFR between LTCF residents and community-dwelling older adults, because the metaregression indicates that IFR triples with each additional 10 years of age.[2] The study of Axfors and Ioannidis (2022), published in this journal, compute IFR estimates for community-dwelling adults that can be directly compared with the metaregression predictions.[72] Here are several notable examples:

- *England*: The estimated IFR is 9·7% for community-dwelling adults ages 70+, which is a cohort with a median age of about 79 years.[72] At this median age, the metaregression predicts an IFR of 7·5% (CI: 6·1–9·2%).
- *France*: The estimated IFR is 4·1% for all French community-dwelling adults ages 65+, which is a cohort with a median age of about 75 years.[73] At this median age, the metaregression predicts an IFR of 4·6% (CI: 3·8–5·6%).
- *USA*: The estimated IFR is 2·3% for U.S. community-dwelling adults ages 65+, although that IFR is 3·6% based on COVID-19 fatalities as reported by the U.S. federal agency that oversees all skilled nursing facilities (rather than from an alternative source that encompasses group homes and assisted living facilities).[11, 74] This age cohort has a median age of 72 years, and the metaregression predicts an IFR of 3·2% (CI: 2·7–3·8%).

For other national studies involving representative samples of community-dwelling older adults, the estimated IFR is about 2% for Andorra and Netherlands, 3% for Denmark, 5% for Spain, and 8% for Italy. The IFR estimate for Hungary is highly uncertain, because its seroprevalence was not distinguishable from zero in light of comprehensive data on assay characteristics.[75, 76] Findings from other national studies -- such as the Belgium study of residual sera, the Canada study of blood donors, the Iceland and Israel studies of healthcare patients, and the USA study of dialysis outpatients -- are based on convenience samples and hence subject to elevated risk of bias.[77–81].

## Lessons for the Future

COVID-19 has had a catastrophic impact on LTCF residents. Some countries, such as France and UK, that had been hit hard by the first wave incurred fewer LTCF fatalities during the second wave, reflecting protective measures as well as the natural immunity of those previously infected.[55, 82] However, the incidence of LTCF fatalities continued rising markedly during the second wave in several other countries (including Denmark, Germany, and USA), reflecting outbreaks at LTCFs that had been left unscathed during the first wave.[83, 84].

More recently, mortality has been sharply reduced by the nearly universal dissemination of vaccines to LTCF residents and staff. For example, the COVID-19 mortality rate of U.S. LTCF residents in March-December 2021 was 780 per 100 K, less than one-tenth of its rate over the preceding 12-month period.[11] Nonetheless, even following booster shots, vaccinated LTCF residents may be particularly prone to so-called “breakthrough infections” due to immune dysregulation and other factors, and the frequency of such cases has increased following the emergence of the Omicron variant of the virus.[82] Thus, it would be premature to conclude that the pandemic no longer poses a substantial threat to LTCF residents.

Numerous studies have found that the risk of COVID-19 spreading within an LTCF is closely linked to the incidence of SARS-Cov-2 infections in the surrounding area.[85–88] Consequently, LTCFs have endeavored to mitigate those risks by implementing non-pharmaceutical interventions such as comprehensive testing, visitor restrictions, and use of masks. However, measures such as visitor restrictions can exacerbate LTCF residents’ sense of isolation, fear, and depression, while the use of protective gear by staff can be particularly disturbing to residents with impaired cognitive or hearing abilities. Systematic reviews have found that social isolation and loneliness in LTCF residents are strongly associated with increased mortality, which is particularly concerning given that about one-third of LTCF residents were already experiencing severe loneliness prior to the pandemic.[89–91].

Most LTCF residents have relatively few close contacts, whereas staff members interact with many residents and with close contacts in the community and hence have a higher risk of conveying the virus into the facility. Many jobs in LTCFs are associated with low pay, and hence staff are more likely to live in high-density neighborhoods and use public transport, and many have family members who are also employed in high-contact service jobs; moreover, LTCF staff are primarily female and hence more likely to have child care responsibilities.[92, 93] And if an staff member becomes ill, no substitute may be available to carry out their duties. At the early stages of the pandemic some LTCF staff took heroic steps, such as moving into the facility to limit their exposure to infection, but such measures are clearly not practical or sustainable in the vast majority of instances.[94, 95].

Looking forward, perhaps the most promising approach for mitigating the risk of infectious diseases in LTCFs might be to create living spaces that are more akin to a family home.[96] As in Denmark, for example, each facility could be subdivided into a smaller set of ‘houses’, each of which has its own dedicated staff.[97] Implementing such an approach would necessarily be a long-term solution that would require careful consideration of funding, staffing, and other factors. Nonetheless, it is imperative for public authorities and academic researchers to engage in “lessons learned” from the COVID-19 pandemic that would have crucial benefits for protecting the health and well-being of LTCF residents.

## References

[1] Buck PS (1954). My Several Worlds: A Personal Record.

[2] Levin AT, Hanage WP, Owusu-Boaitey N, Cochran KB, Walsh SP, Meyerowitz-Katz G (2020). Assessing the age specificity of infection fatality rates for COVID-19: systematic review, meta-analysis, and public policy implications. Eur J Epidemiol.

[3] Pifarré i Arolas H, Acosta E, López-Casasnovas G (2021). Years of life lost to COVID-19 in 81 countries. Sci Rep.

[4] Aburto JM, Schöley J, Kashnitsky I (2021). Quantifying impacts of the COVID-19 pandemic through life-expectancy losses: a population-level study of 29 countries. Int J Epidemiol.

[5] Norway Institute of Public Health (FHI). Daily report and statistics about coronavirus and COVID-19. 2021.

[6] Sweden Public Health Agency. COVID-19 Statistics. 2021.

[7] Scotland National Records (NRS). Deaths involving Coronavirus (COVID-19) in Scotland. 2021.

[8] U.K. Office for National Statistics (ONS). Deaths involving COVID-19 in the care sector, England and Wales. 2021.

[9] U.K. Office for National Statistics (ONS). Coronavirus (COVID-19) Data. 2021.

[10] U.S. Center for Disease Control & Prevention. COVID Data Tracker. 2021.

[11] U.S. Center for Medicare & Medicaid Services (CMS). COVID-19 Nursing Home Data. 2021.

[12] France National Institute for Demographic Studies (INED). The Demography of COVID-19 Deaths: Cumulative Fatalities by Age and Place of Death. 2022.

[13] Islam N, Jdanov DA, Shkolnikov VM (2021). Effects of covid-19 pandemic on life expectancy and premature mortality in 2020: time series analysis in 37 countries. BMJ.

[14] Quast T, Andel R, Gregory S, Storch EA (2021). Years of life lost associated with COVID-19 deaths in the USA during the first year of the pandemic. J Public Health.

[15] Albertini M, Gähler M, Härkönen J (2018). Moving back to “mamma”? Divorce, intergenerational coresidence, and latent family solidarity in Sweden. Popul Space Place.

[16] Ballin M, Bergman J, Kivipelto M, Nordström A, Nordström P (2021). Excess Mortality After COVID-19 in Swedish Long-Term Care Facilities. J Am Med Dir Assoc.

[17] Candel FJ, Barreiro P, San Román J (2021). The demography and characteristics of SARS-CoV-2 seropositive residents and staff of nursing homes for older adults in the Community of Madrid: the SeroSOS study. Age Ageing.

[18] Chudasama DY, Milbourn H, Nsonwu O (2021). Penetration and impact of COVID-19 in long term care facilities in England: population surveillance study. Int J Epidemiol.

[19] Sweden Institute. Elderly Care in Sweden. 2022.

[20] World Health Organization. Data Portal on Ageing. 2022.

[21] Statistics USNCfH. Long-Term Care Providers and Service Users in the United States, 2015–2016. 2019.

[22] Kyu HH, Abate D, Abate KH, et al. Global, regional, and national disability-adjusted life-years (DALYs) for 359 diseases and injuries and healthy life expectancy (HALE) for 195 countries and territories, 1990-2017: a systematic analysis for the Global Burden of Disease Study 2017. The Lancet. 2018;392(10159):1859–922. doi:10.1016/S0140-6736(18)32335-3. 10.1016/S0140-6736(18)32335-3 PMC6252083 30415748

[23] OECD Health Statistics. Definitions, Sources, and Methods: Beds in residential long-term care facilities. 2021.

[24] Katz MJ (2021). Caring for those caring for older adults. The Lancet Healthy Longevity.

[25] OECD. Elderly population. 2022.

[26] Brändström A, Meyer AC, Modig K, Sandström G (2021). Determinants of home care utilization among the Swedish old: nationwide register-based study. Eur J Ageing.

[27] OECD. Public Long-term Care Financing Arrangements in OECD Countries. 2011.

[28] Jolanki OH. Senior Housing as a Living Environment That Supports Well-Being in Old Age. Front Public Health. 2021;8:1–12. doi:10.3389/fpubh.2020.589371. 10.3389/fpubh.2020.589371 PMC7890193 33614564

[29] Collard RM, Boter H, Schoevers RA, Oude Voshaar RC (2012). Prevalence of Frailty in Community-Dwelling Older Persons: A Systematic Review. J Am Geriatr Soc.

[30] Veronese N, Custodero C, Cella A (2021). Prevalence of multidimensional frailty and pre-frailty in older people in different settings: A systematic review and meta-analysis. Ageing Res Rev.

[31] U.S. National Center for Health Statistics. Long-term Care Providers and Services Users in the United States, 2015–2016. 2019;Series 3, Number 3. 27023287

[32] Todd S, Barr S, Roberts M, Passmore AP (2013). Survival in dementia and predictors of mortality: a review. Int J Geriatr Psychiatry.

[33] Arrighi HM, Neumann PJ, Lieberburg IM, Townsend RJ (2010). Lethality of Alzheimer Disease and Its Impact on Nursing Home Placement. Alzheimer Disease & Associated Disorders.

[34] Forder J, Fernandez J-L. Length of stay in care homes. PSSRU Discussion Paper 2769. 2011.

[35] Vena A, Berruti M, Adessi A (2020). Prevalence of Antibodies to SARS-CoV-2 in Italian Adults and Associated Risk Factors. J Clin Med.

[36] Biggs HM. Estimated Community Seroprevalence of SARS-CoV-2 Antibodies — Two Georgia Counties, April 28–May 3, 2020. MMWR Morb Mortal Wkly Rep. 2020;69(29):965–70. doi:10.15585/MMWR.MM6929E2. 10.15585/mmwr.mm6929e2 PMC7377817 32701941

[37] Telford C, Onwubiko U, Holland D, et al. Preventing COVID-19 Outbreaks in Long-Term Care Facilities Through Preemptive Testing of Residents and Staff Members — Fulton County, Georgia, March–May 2020. MMWR Morb Mortal Wkly Rep 2020;69. doi:10.15585/mmwr.mm6937a4. 10.15585/mmwr.mm6937a4 PMC7498169 32941413

[38] Dutey-Magni PF, Williams H, Jhass A (2021). COVID-19 infection and attributable mortality in UK care homes: cohort study using active surveillance and electronic records (March–June 2020). Age Ageing.

[39] Konetzka RT, White EM, Pralea A, Grabowski DC, Mor V (2021). A systematic review of long-term care facility characteristics associated with COVID-19 outcomes. J Am Geriatr Soc.

[40] Tao J, Zhang X, Musa SS, Yang L, He D. High Infection Fatality Rate Among Elderly and Risk Factors Associated With Infection Fatality Rate and Asymptomatic Infections of COVID-19 Cases in Hong Kong. Front Med. 2021;8:1–8. doi:10.3389/fmed.2021.678347. 10.3389/fmed.2021.678347 PMC8180593 34109200

[41] Hashan MR, Smoll N, King C, et al. Epidemiology and clinical features of COVID-19 outbreaks in aged care facilities: A systematic review and meta-analysis. eClinicalMedicine. 2021;33:1–2. doi:10.1016/j.eclinm.2021.100771. 10.1016/j.eclinm.2021.100771 PMC7917447 33681730

[42] Burton JK, Bayne G, Evans C (2020). Evolution and effects of COVID-19 outbreaks in care homes: a population analysis in 189 care homes in one geographical region of the UK. The Lancet Healthy Longevity.

[43] Fisman DN, Bogoch I, Lapointe-Shaw L, McCready J, Tuite AR (2020). Risk Factors Associated With Mortality Among Residents With Coronavirus Disease 2019 (COVID-19) in Long-term Care Facilities in Ontario, Canada. JAMA Netw Open.

[44] World Health Organization. International Guidelines for Certification and Classification (Coding) of COVID-19 as Cause of Death. 2020.

[45] U.K. Office for National Statistics (ONS). Monthly mortality analysis, England and Wales, Table 12. 2021.

[46] Sweden National Board. of Health & Welfare (Socialstyrelsen). COVID-19 statistics. 2021.

[47] Edler C, Schröder AS, Aepfelbacher M (2020). Dying with SARS-CoV-2 infection—an autopsy study of the first consecutive 80 cases in Hamburg, Germany. Int J Legal Med.

[48] Elezkurtaj S, Greuel S, Ihlow J (2021). Causes of death and comorbidities in hospitalized patients with COVID-19. Sci Rep.

[49] Danics K, Pesti A, Törő K (2021). A COVID-19-association-dependent categorization of death causes in 100 autopsy cases. GeroScience.

[50] Romanova ES, Vasilyev VV, Startseva G, Karev V, Rybakova MG, Platonov PG (2021). Cause of death based on systematic post-mortem studies in patients with positive SARS-CoV-2 tissue PCR during the COVID-19 pandemic. J Intern Med.

[51] De-Giorgio F, Grassi VM, Bergamin E (2021). Dying “from” or “with” COVID-19 during the Pandemic: Medico-Legal Issues According to a Population Perspective. Int J Environ Res Public Health.

[52] Esteban I, Bergero G, Alves C, et al. Asymptomatic COVID-19 in the elderly: dementia and viral clearance as risk factors for disease progression. Gates Open Research. 2021;5:1–12. 10.12688/gatesopenres.13357.1 PMC8987012 35441127

[53] Kamerlin SCL, Kasson PM (2020). Managing Coronavirus Disease 2019 Spread With Voluntary Public Health Measures: Sweden as a Case Study for Pandemic Control. Clin Infect Dis.

[54] Vogel G (2020). Sweden’s Gamble. Science.

[55] Krutikov M, Palmer T, Tut G (2021). Incidence of SARS-CoV-2 infection according to baseline antibody status in staff and residents of 100 long-term care facilities (VIVALDI): a prospective cohort study. The Lancet Healthy Longevity.

[56] International Long-Term Care Policy Network. COVID-19 mortality and long-term care: a UK comparison. 2020.

[57] Brown KA, Daneman N, Buchan SA, Chan AK, Stall NM (2021). Variation in Care of Community and Nursing Home Residents Who Died of COVID-19 in Ontario, Canada. J Am Med Dir Assoc.

[58] Bernadou A, Bouges S, Catroux M (2021). High impact of COVID-19 outbreak in a nursing home in the Nouvelle-Aquitaine region, France, March to April 2020. BMC Infect Dis.

[59] Murti M, Goetz M, Saunders A (2021). Investigation of a severe SARS-CoV-2 outbreak in a long-term care home early in the pandemic. Can Med Assoc J.

[60] Akhtar-Danesh N, Baumann A, Crea-Arsenio M, Antonipillai V (2022). COVID-19 excess mortality among long-term care residents in Ontario, Canada. PLoS ONE.

[61] Lee DS, Ma S, Chu A (2021). Predictors of mortality among long-term care residents with SARS-CoV-2 infection. J Am Geriatr Soc.

[62] Chao C-T, Lee S-Y, Wang J, Chien K-L, Huang J-W (2021). Frailty increases the risk for developing urinary tract infection among 79,887 patients with diabetic mellitus and chronic kidney disease. BMC Geriatr.

[63] Iwai-Saito K, Shobugawa Y, Aida J, Kondo K (2021). Frailty is associated with susceptibility and severity of pneumonia in older adults (A JAGES multilevel cross-sectional study). Sci Rep.

[64] Knopp P, Miles A, Webb TE (2020). Presenting features of COVID-19 in older people: relationships with frailty, inflammation and mortality. Eur Geriatr Med.

[65] Wang GC, Casolaro V (2014). Immunologic changes in frail older adults. Transl Med UniSa.

[66] Zhang X-M, Jiao J, Cao J (2021). Frailty as a predictor of mortality among patients with COVID-19: a systematic review and meta-analysis. BMC Geriatr.

[67] Bielza R, Sanz J, Zambrana F (2021). Clinical Characteristics, Frailty, and Mortality of Residents With COVID-19 in Nursing Homes of a Region of Madrid. J Am Med Dir Assoc.

[68] Shi SM, Bakaev I, Chen H, Travison TG, Berry SD (2020). Risk Factors, Presentation, and Course of Coronavirus Disease 2019 in a Large, Academic Long-Term Care Facility. J Am Med Dir Assoc.

[69] Mak JKL, Kuja-Halkola R, Wang Y, Hägg S, Jylhävä J (2021). Frailty and comorbidity in predicting community COVID-19 mortality in the U.K. Biobank: The effect of sampling. J Am Geriatr Soc.

[70] Panagiotou OA, Kosar CM, White EM (2021). Risk Factors Associated With All-Cause 30-Day Mortality in Nursing Home Residents With COVID-19. JAMA Intern Med.

[71] Aliberti MJR, Avelino-Silva TJ (2021). Beyond Age—Improvement of Prognostication Through Physical and Cognitive Functioning for Nursing Home Residents With COVID-19. JAMA Intern Med.

[72] Axfors C, Ioannidis J. Infection Fatality Rate of COVID-19 in Community-Dwelling Elderly Populations. European Journal of Epidemiology. 2022. 10.1007/s10654-022-00853-w PMC8934243 35306604

[73] PopulationPyramid.net. Population Pyramid for France. 2021.

[74] Kaiser Family Foundation. This Week in Coronavirus: June 18 to June 25. 2020.

[75] Merkely B, Szabo AJ, Kosztin A (2020). Novel coronavirus epidemic in the Hungarian population, a cross-sectional nationwide survey to support the exit policy in Hungary. Geroscience.

[76] Pollán M, Pérez-Gómez B, Pastor-Barriuso R (2020). Prevalence of SARS-CoV-2 in Spain (ENE-COVID): a nationwide, population-based seroepidemiological study. The Lancet.

[77] Anand S, Montez-Rath M, Han J, et al. Prevalence of SARS-CoV-2 antibodies in a large nationwide sample of patients on dialysis in the USA: a cross-sectional study. The Lancet. 2020. doi:10.1016/S0140-6736(20)32009-2. 10.1016/S0140-6736(20)32009-2 PMC7518804 32987007

[78] Gudbjartsson DF, Norddahl GL, Melsted P (2020). Humoral Immune Response to SARS-CoV-2 in Iceland. N Engl J Med.

[79] Herzog S, Bie JD, Abrams S, et al. Seroprevalence of IgG antibodies against SARS coronavirus 2 in Belgium: a prospective cross-sectional study of residual samples. Euro Surveillance. 2022;27.10.2807/1560-7917.ES.2022.27.9.2100419. 10.2807/1560-7917.ES.2022.27.9.2100419 PMC8895468 35241216

[80] Saeed S, Drews SJ, Pambrun C, Yi Q-L, Osmond L, O’Brien SF (2021). SARS-CoV-2 seroprevalence among blood donors after the first COVID-19 wave in Canada. Transfusion.

[81] Reicher S, Ratzon R, Ben-Sahar S (2021). Nationwide seroprevalence of antibodies against SARS-CoV-2 in Israel. Eur J Epidemiol.

[82] Seiffert P, Konka A, Kasperczyk J, et al. Immunogenicity of the BNT162b2 mRNA COVID-19 vaccine in older residents of a long-term care facility: relation with age, frailty and prior infection status. Biogerontology. 2021;23:1–12. doi:10.1007/s10522-021-09944-9. 10.1007/s10522-021-09944-9 PMC8684786 34923608

[83] Heneghan C, Dietrich M, Brassey J, Jefferson T, Kay A. CG Report 6: Effects of COVID-19 in Care Homes - A Mixed Methods Review. 2021.

[84] Ioannidis JPA, Axfors C, Contopoulos-Ioannidis DG (2021). Second versus first wave of COVID-19 deaths: Shifts in age distribution and in nursing home fatalities. Environ Res.

[85] Gorges RJ, Konetzka RT (2020). Staffing Levels and COVID-19 Cases and Outbreaks in U.S. Nursing Homes. J Am Geriatr Soc.

[86] Shallcross L, Burke D, Abbott O (2021). Factors associated with SARS-CoV-2 infection and outbreaks in long-term care facilities in England: a national cross-sectional survey. The Lancet Healthy Longevity.

[87] White EM, Kosar CM, Feifer RA (2020). Variation in SARS-CoV-2 Prevalence in U.S. Skilled Nursing Facilities. J Am Geriatr Soc.

[88] Sepulveda ER, Stall NM, Sinha SK (2020). A Comparison of COVID-19 Mortality Rates Among Long-Term Care Residents in 12 OECD Countries. J Am Med Dir Assoc.

[89] Gardiner C, Laud P, Heaton T, Gott M (2020). What is the prevalence of loneliness amongst older people living in residential and nursing care homes? A systematic review and meta-analysis. Age Ageing.

[90] Leigh-Hunt N, Bagguley D, Bash K (2017). An overview of systematic reviews on the public health consequences of social isolation and loneliness. Public Health.

[91] Rico-Uribe LA, Caballero FF, Martín-María N, Cabello M, Ayuso-Mateos JL, Miret M (2018). Association of loneliness with all-cause mortality: A meta-analysis. PLoS ONE.

[92] OECD. Long-term care workers. 2019.

[93] True S, Cubanski J, Garfield R, et al. COVID-19 and Workers at Risk: Examining the Long-Term Care Workforce. Kaiser Family Foundation COVID-19 Issue Brief. 2020.

[94] BBC staff. Coronavirus: Staff ‘saved lives’ by moving into Doncaster care home. BBC News. 2020 14 May 2020.

[95] Murray J. ‘We did what we set out to achieve’: the staff who moved into care homes. The Guardian. 2020 28 April 2020.

[96] Heckman GA, Kay K, Morrison A, et al. Proceedings from an International Virtual Townhall: Reflecting on the COVID-19 Pandemic: Themes from Long-Term Care. Journal of the American Medical Directors Association. 2021;22(6):1128-32. doi:10.1016/j.jamda.2021.03.029. 10.1016/j.jamda.2021.03.029 PMC8030741 33932351

[97] European Centre for Social Welfare Policy and Research. Denmark: Social housing for older people in the Act on Social Housing. 2016.

